# Current Trends in the Management of Epithelial Lacrimal Gland Tumors: A Retrospective National Cancer Database Analysis

**DOI:** 10.7759/cureus.27109

**Published:** 2022-07-21

**Authors:** Prashanth Ashok Kumar, Dongliang Wang, Danning Huang, Shweta Paulraj, Abirami Sivapiragasam

**Affiliations:** 1 Hematology-Oncology, Upstate University Hospital, Syracuse, USA; 2 Public Health and Preventive Medicine, Upstate University Hospital, Syracuse, USA; 3 Internal Medicine and Cardiology, Upstate University Hospital, Syracuse, USA

**Keywords:** ncdb, intra-arterial cytoreductive chemotherapy, orbit sparing surgery, orbital exenteration, lacrimal gland tumor

## Abstract

Background: Lacrimal gland tumors are rare with data limited to very few large studies. Contemporary strategies like orbit sparing surgeries and neoadjuvant intraarterial chemotherapy remain controversial.

Methods: This is a retrospective cohort analysis of epithelial lacrimal gland tumors from the 2004-2016 National Cancer Database. Patients were stratified based on the type of surgery (limited vs destructive) and various treatment modalities employed.

Results: Squamous cell carcinoma (33.48%) and adenoid cystic carcinoma (29.45%) were the commonest histologies (N=669). Comparison of limited (46.33%) vs destructive procedures (53.11%) among 482 patients did not show any survival difference, nor the comparison between surgery vs ± chemotherapy vs ± radiotherapy among 472 patients.

Conclusion: Squamous cell carcinoma and adenoid cystic carcinoma are the commonest types of lacrimal gland tumors seen in our study. Tumor spread from adjacent sites may have contributed to the higher percentage of squamous cell carcinomas seen. The type of surgery or chemoradiation use did not alter survival.

## Introduction

The lacrimal gland is a small pear-shaped organ in the orbit and can give rise to malignancies with significant morbidity and mortality. Tumors from this hidden yet clinically significant organ have demonstrated rapid dissemination through the surrounding bones providing considerable challenges to care providers [1]. The incidence of lacrimal gland tumors is around one in 1,000,000 per year. Epithelial lesions are the most common (>50%), followed by lymphoid lesions (around 25%), with the remaining being mesenchymal and metastatic lesions (10-15%) [2]. The estimated five-year mortality is around 50%, which shows the aggressive nature of the disease. The management of lacrimal gland tumors is very heterogeneous with no standard guideline. The optimal strategy to be employed remains controversial and is a subject of debate among academia [1,3,4]. Orbital exenteration with or without the removal of the bony walls has been the gold standard surgical approach practiced for decades [2]. With the dawn of modern oncology and personalized medicine, more and more providers are practicing approaches like orbital sparing procedures, postoperative radiation, and intra-arterial cytoreductive chemotherapy (IACC). Despite their sporadic use, data on the effect of these novel strategies on survival and outcomes is very scarce with no major prospective clinical trials [1,5,6]. We hope to fill this void using the National Cancer Database (NCDB), a very large national hospital database that has been widely used in oncology [7]. Our aim was to use the NCDB data to analyze the demographic, histopathology, and clinical features of patients with lacrimal gland tumors and to study the difference in outcome between patients undergoing radical and limited surgical procedures. Additionally, we wanted to compare the outcomes between different adjuvant and neoadjuvant strategies used for lacrimal gland tumors. 

## Materials and methods

The NCDB is a very large repository managed by the American College of Surgeons and has been used in several cancer-related studies. The database contains information on patient demographics, comorbidities, tumor characteristics, treatment information, and mortality [7,8]. The State University of New York (SUNY) Upstate Institutional Review Board (IRB) reviewed the project (approval 1495194-1) and determined that the project does not meet the definition of human subject research. 

From the 2004-2016 Participant User File (PUF) file for Head and Neck cancers, we selected patients who had the code for lacrimal gland tumors. The code used was “c69.5”, which is the International Classification for Diseases of Oncology (ICD-O-3) code for lacrimal gland primary site [9]. We included epithelial malignancies arising primarily from the lacrimal glands (carcinoma, carcinoma undifferentiated, papillary carcinoma, squamous cell carcinoma (SCC), lymphoepithelial carcinoma, transitional cell carcinoma, papillary transitional cell carcinoma, adenocarcinoma, adenoid cystic carcinoma (ACC), cribriform carcinoma, bronchioloalveolar carcinoma, oxyphilic adenocarcinoma, mucoepidermoid carcinoma, and carcinoma ex pleomorphic adenoma. The histologies listed (Table 1) were queried and formed the cohort for subsequent analysis. ICO-O-3 histology codes were used for the same [10]. The type of the most definitive surgical procedure used was analyzed. Patients who received hormone or immunotherapy and those who were considered for or had an unknown status of hormonal therapy or immunotherapy use were not included. This was done by including only those who did not definitively receive hormone or immunotherapy. This was done so that the effects of chemotherapy (CT) and radiation therapy (RT) could be analyzed and are not confounded by the use of hormone or immunotherapy. Patients who did not undergo any surgical procedure or had a debulking procedure and those who had uncertain or unknown values were excluded. We divided the above cohort into those undergoing radical and destructive procedures and those undergoing limited or orbit sparing procedures. The former included total removal of the primary site, ie, enucleation and radical surgery. The limited group included local tumor destruction not otherwise specified (NOS), photodynamic therapy (PDT), electrocautery, fulguration (includes use of hot forceps for tumor destruction), cryosurgery, laser, local tumor excision NOS, polypectomy and excisional biopsy. It also included the combination of local tumor excision NOS, polypectomy, and excisional biopsy with PDT, electrocautery, cryosurgery, laser ablation, or laser excision. Simple/partial surgical removal of primary site was also a part of the limited group. The subset obtained was further subdivided based on the various strategies of CT and RT used. Patients who did not receive CT or RT or had an unknown status were excluded.

**Table 1:** Histological subtypes and their distribution seen among lacrimal gland tumors (N=669) NOS: not otherwise specified; SCC: squamous cell carcinoma; ACC: adenoid cystic carcinoma

Main Histologic Type	N (%)	Histologic Subtype	N (%)
Carcinoma, NOS Carcinoma, Undifferentiated, NOS	46 (6.88%)	Carcinoma, NOS	38 (5.68%)
		Large cell carcinoma, NOS	1 (0.15%)
		Carcinoma undifferentiated type, NOS	2 (0.3%)
		Pleomorphic carcinoma	5 (0.75%)
Papillary Carcinoma, NOS	11 (1.64%)	Papillary carcinoma, NOS	1 (0.15%)
		Papillary squamous cell carcinoma	10 (1.49%)
SCC, NOS	224 (33.48%)	SCC, NOS	185 (27.65%)
		SCC Keratinizing, NOS	14 (2.09%)
		SCC, Ig, cell, Non-keratinizing	25 (3.74%)
Lymphoepithelial Carcinoma	22 (3.29%)	Lymphoepithelial carcinoma	3 (0.45%)
		Basaloid Squamous cell carcinoma	19 (2.84%)
Transitional Cell Carcinoma, NOS	20 (2.99%)	Transitional cell carcinoma	14 (2.09%)
		Schneiderian carcinoma	2 (0.3%)
		Basaloid carcinoma	4 (0.6%)
Papillary Transitional Cell Carcinoma	6 (0.9%)		6 (0.9%)
Adenocarcinoma, NOS	72 (10.76%)	Adenocarcinoma, NOS	70 (10.46%)
		Basal cell adenocarcinoma	2 (0.3%)
ACC	197 (29.45%)		197 (29.45%)
Cribriform carcinoma	1 (0.15%)		1 (0.15%)
Broncho-alveolar adeno carcinoma	2 (0.3%)		2 (0.3%)
Oxyphilic Adenocarcinoma	7 (1.05%)		7 (1.05%)
Mucoepidermoid Carcinoma	49 (7.32%)		49 (7.32%)
Carcinoma in pleomorphic adenoma	12 (1.79%)		12 (1.79%)

**Figure 1:**
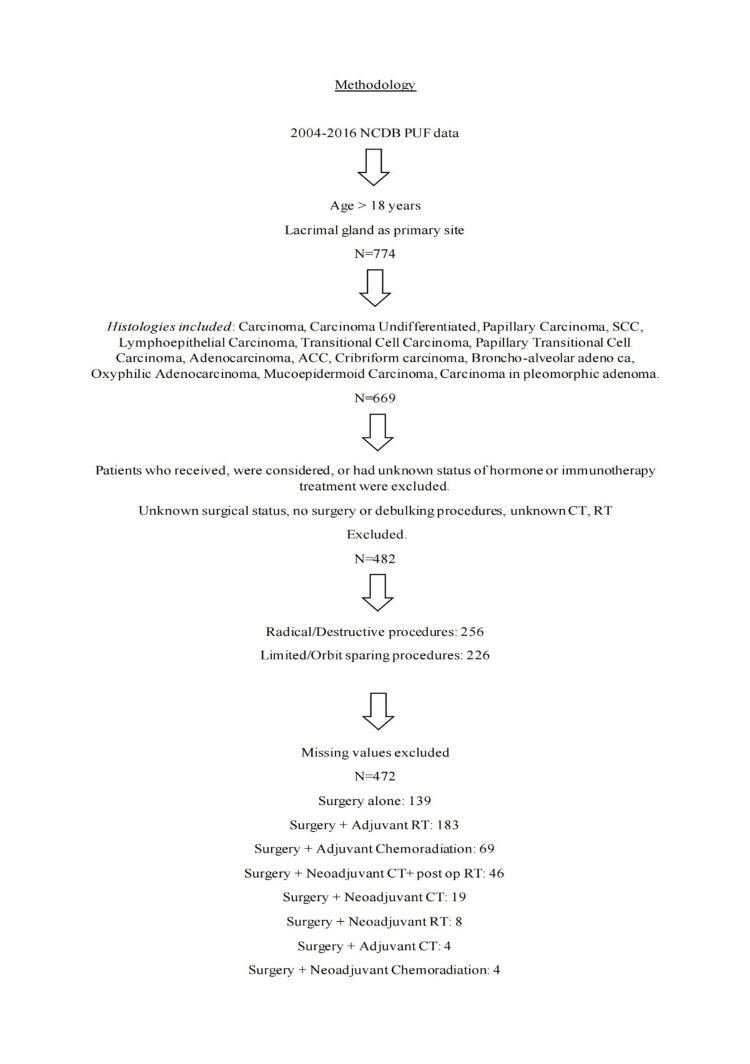
Summary and outline of methodology and patient population NCDB: National Cancer Database; PUF: participant user file; SCC: squamous cell carcinoma; ACC: adenoid cystic carcinoma; CT: chemotherapy; RT: radiation therapy

Count and proportion were used to summarize the distribution of variables. Kaplan-Meier survival analysis and point estimates were performed to assess the survival difference between different groups. No multivariate analysis was further performed, given no statistical significance is observed from univariate analysis. The methodical flow of the study has been depicted in Figure 1.

## Results

A total of 774 lacrimal gland tumors were found in the NCDB PUF data from 2004-2016. We searched for the histological subtypes listed (Table 1), which gave us a cohort of 669 patients. SCC (33.48%) was the most common followed by ACC (29.45%), adenocarcinoma, NOS (10.76%), and mucoepidermoid carcinoma (7.32%). The demographic and pathological characteristics of the final cohort are summarized in Table 2.

**Table 2:** Demographic, clinical, and pathological characteristics of the study population NOS: not otherwise specified.

Age in years (N=482)	Distribution (%)
<65	300 (62.24)
>=65	182 (39.73)
Race (N=482)	
Caucasian	375 (77.8)
African American	62 (12.86)
Others	45 (9.34)
Type of Facility (N=415, Missing=67)	
Academic	331 (79.76)
Community	84 (20.24)
Area of Residence 2013 (N=482)	
Metro	408 (84.65)
Urban	74 (15.35)
Median Income in dollars from 2008-2012 (N=481, Missing=1)	
<48000	189 (39.29)
>48000	292 (60.71)
Insurance type (N=482)	
Uninsured	44 (9.13)
Private	232 (48.13)
Government	206 (42.74)
Percentage of people in the area of residence who have completed high school degrees from 2008-2012 (N=481, Missing=1)	
>=21	88 (18.30)
13-20.9	117 (24.32)
7-12.9	157 (32.64)
<7	119 (24.74)
Charlson Deyo Score (N=482)	
0	392 (81.33)
1	74 (15.35)
2 or 3	16 (3.32)
Tumor Grade (N=482)	
Well differentiated, differentiated, NOS	33 (6.85)
Moderately differentiated, moderately well differentiated, intermediate differentiation	98 (20.33)
Poorly differentiated	134 (27.8)
Undifferentiated, anaplastic	16 (3.32)
Cell type not determined, not stated or not applicable, unknown primaries, high grade dysplasia	201 (41.7)

**Figure 2:**
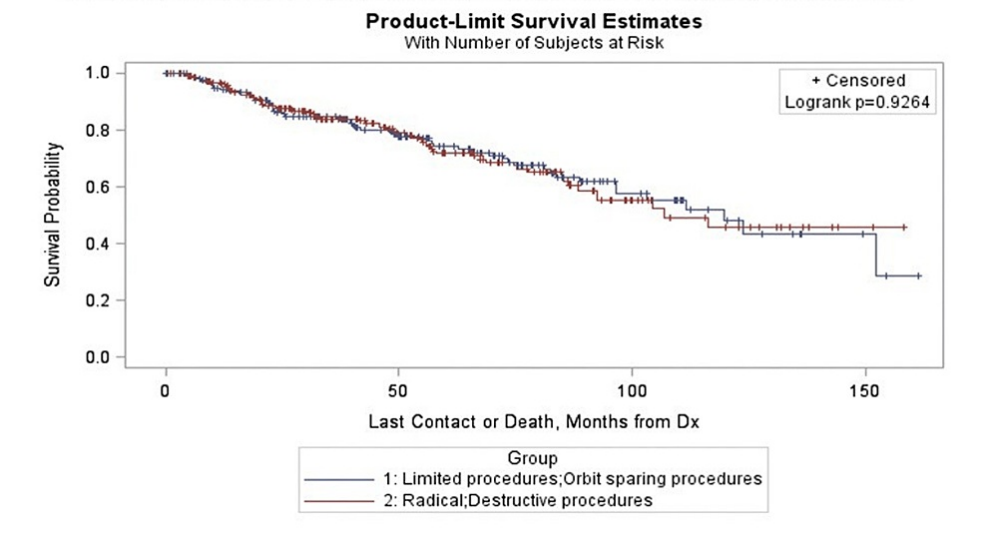
Kaplan-Meier survival curves in months for limited and radical surgical procedures Dx: diagnosis

**Figure 3:**
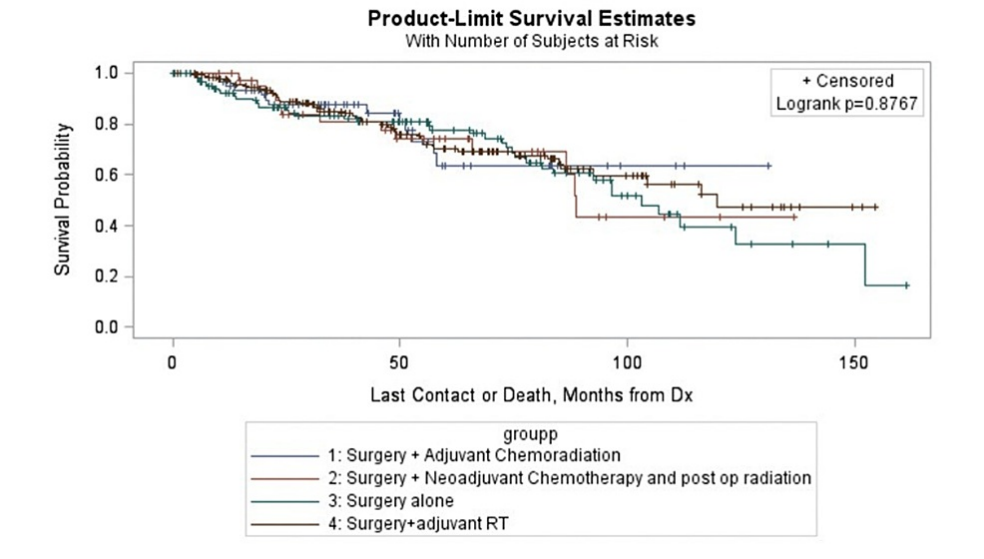
Kaplan-Meier survival curves in months for the various combinitions of adjuvant and neoadjuvant chemotherapy and radiation therapy RT: radiation therapy; Dx: diagnosis

The frequency distribution of the various surgical procedures done is represented in Table 3. The procedures were grouped into radical/destructive procedures and limited/orbit sparing procedures. Of the patients, 53.11% underwent a radical procedure, whereas 46.89% had a limited orbit sparing procedure. Based on the combinations of adjuvant or neoadjuvant CT or RT used, patients were divided into eight groups (Table 3). The distribution between the various treatment groups and subgroups, five-year and 10-year survival estimates based on KM analysis, and the "head-on" survival comparison based on adjusted cox model with 95% wald limits are represented in Table 3. Surgery + adjuvant RT (38.77%) was the most frequently used treatment modality followed by surgery alone (29.45%) and surgery + adjuvant chemoradiation (14.62%). The KM survival curves in months for the surgical groups (Figure 2) and the treatment subgroups (Figure 3) do not show any significant difference in survival with a P-value of 0.9264 and 0.8767, respectively. The point survival estimates between the various treatment subgroups showed no significant difference in treatment outcomes (Table 3).

**Table 3:** Distribution, 10-year survival estimates, and point survival estimates based on cox models among the groups and subgroups of the final cohort. NOS: not otherwise specified; KM: Kaplan-Meier; CI: confidence interval; CT: chemotherapy; RT: radiation therapy.

Surgical Procedure	Distribution N (%)
Radical/destructive procedures	256 (53.11%)
Total surgical removal of primary site	118 (24.48
Radical surgery	76 (15.77%)
Total enucleation	62 (12.86%)
Limited procedures/orbit sparing procedures	226 (46.89%)
Simple/partial surgical removal of primary site	115 (23.86%)
Excisional biopsy	66 (13.69%)
Local tumor excision, NOS	43 (8.92%)
Any combination of local tumor excision, polypectomy or excisional biopsy with cryosurgery	1 (0.21%)
Any combination of local tumor excision, polypectomy or excisional biopsy with electrocautery	1 (0.21%)

Type of Surgery (N=482)		Survival based on KM analysis with CI			N (%)
		5 years	10 years		
Radical/destructive procedures		0.718 (0.640-0.781)	0.46 (0.331-0.580)		256 (53.11%)
Limited procedures/orbit sparing procedures		0.741 (0.663-0.804)	0.48 (0.343-0.605)		230 (46.89%)
Limited vs radical surgeries; Point estimate, 95% Wald CI (Lower-Upper)		0.983 (0.689-1.404)			
Group No.	Treatment strategy (N=472, Missing=10)	Survival based on KM analysis with CI			Distribution N (%)
		5 years		10 years	
1	Surgery alone	0.778 (0.680-0.850)		0.395 (0.235-0.551)	139 (29.45%)
2	Surgery + Adjuvant RT	0.702 (0.611-0.775)		0.474 (0.317-0.616)	183 (38.77%)
3	Surgery + Adjuvant Chemoradiation	0.635 (0.429-0.784)		0.635 (0.429-0.784)	69 (14.62%)
4	Surgery + Neoadjuvant CT+ post op RT	0.744 (0.564-0.859)		0.432 (0.184-0.660)	46 (9.75%)
5	Surgery + Neoadjuvant CT	0.822 (0.543-0.939)		0.822 (0.543-0.939)	19 (4.03%)
6	Surgery + Neoadjuvant RT	0.667 (0.054-0.945)		0.333 (0.009-0.774)	8 (1.69%)
7	Surgery + Adjuvant CT	-		-	4 (0.85%)
8	Surgery + Neoadjuvant Chemoradiation	-		-	4 (0.85%)
Point estimate, 95% Wald CI (Lower-Upper)					
Surgery alone vs Surgery + Adjuvant RT		0.949 (0.565-1.594)			
Surgery + Adjuvant Chemoradiation vs Surgery + Adjuvant RT		2.166 (0.974-4.817)			
Surgery + Adjuvant Chemoradiation vs Surgery alone		2.284 (1.007-5.183)			
Surgery + Adjuvant Chemoradiation vs Surgery + Neoadjuvant CT and post op RT		1.216 (0.458-3.228)			
Surgery + Neoadjuvant CT and post op radiation vs Surgery + Adjuvant RT		1.782 (0.827-3.841)			
Surgery + Neoadjuvant CT and post op RT vs Surgery alone		1.878 (0.843-4.185)			

## Discussion

The ideal treatment strategy for lacrimal gland cancers is unspecified and can vary based on several factors like histopathology. The information available on these tumors is mainly based on case reports, single-institution studies, and database analysis, with no large lacrimal gland-specific clinical trials [1,11,12,13,14]. Several reports have shown ACC as the most common subtype of malignant lacrimal gland tumors [11]. An analysis using the Surveillance, Epidemiology, and End Results (SEER) database from 1973 to 2010 identified 321 nonlymphoid lacrimal gland tumors, after excluding 433 lymphomas. ACC (32.1%) was the most common histological subtype, followed by SCC (29.9%), mucoepidermoid carcinoma (8.7%), and adenocarcinoma (8.4%) [1]. Although SCC was the most common variant in our study, the percentage of SCC and ACC was not very different from the above-referenced analysis.

Providers undertake an aggressive approach to management with hopes to achieve local control and stop perineural invasion and distant metastasis. Orbital exenteration with or without bony wall excision is a destructive and exhaustive procedure and its benefit in all lacrimal gland tumors is controversial and questionable [12]. Wright et al. performed a single institutional review of 50 malignant lacrimal gland tumor patients and analyzed their outcomes. Local tumor removal by lateral orbitotomy and en bloc resection of orbital contents were done based on the tumor characteristics and the majority of the patients received RT. Outcomes of 35 patients with ACC were studied. They were divided into groups based on treatment, which included RT alone (14), local resection and RT (10), and cranio-orbital resection with or without RT (11). Longer time to recurrence and better survival was noted with local resection and RT compared to RT alone. No significance was seen when cranio-orbital resection with or without RT was compared with local resection with RT, leading to the hypothesis that the latter may be as effective as the former [12]. The small sample size was a major limitation of the analysis [6,12]. In a review of seven lacrimal gland tumors treated with destructive surgeries and RT, five died at 12-32 months due to distant metastasis and two remained alive after 24 months [13]. Despite the increasing use of orbital sparing procedures, data on the same is very limited. Esmaeli et al. reported 11 patients who had globe-sparing surgeries of which, 10 had adjuvant RT and six had concurrent chemoradiation. All of them remained disease free at a median follow-up of 33 months [14]. Mallen-St Clair et al. using the SEER database showed that RT improves overall survival in SCC but not in ACC, whereas surgery improves survival in both SCC and ACC [1].

Neoadjuvant chemotherapeutic approaches first came to light in 1998 when Meldrum et al. reported two cases of lacrimal gland tumors with neoadjuvant intracarotid cisplatin and intravenous doxorubicin prior to orbital exenteration. Both the patients achieved radiographic tumor shrinkage and had long-term survival. It was believed that administering a therapeutic agent through this route resulted in a higher concentration and better therapeutic index of the drug at the target site [15]. Intra-arterial administration can also negate the molecular adaptive mechanisms employed by tumor cells, making them more responsive to therapy than traditional therapy. The drug is administered via the external carotid artery, which gives direct branches to the lacrimal gland and not the internal carotid artery, to prevent perfusion to the brain. The risk of toxicity is also reduced when compared to systemic chemotherapy [16]. Tse et al. treated 19 patients with lacrimal gland tumors with this novel modality. Eight had an intact lacrimal artery and the remaining had the tumor and gland resected prior to the intra-arterial chemotherapy. They gave two to three cycles of intra-arterial cisplatin and IV doxorubicin, orbital exenteration, chemoradiation, and three to four further cycles of systemic chemotherapy unless poorly tolerated by the patient. The test cohort was compared with 16 conventionally treated patients. ACC was the predominant histologic subtype. Patients with an intact lacrimal artery had double the 10-year disease-free survival as those without the artery. Both the groups had much better outcomes compared to the conventional group [17]. There are several small reports demonstrating the benefit of this mode of therapy, however, all the available reports have a small sample size lacking sufficient power. Lack of randomization is another drawback of the available data [16]. New frontiers are emerging, which may shape future management strategies like the Fibroblast Growth Factor Receptor 1 Inhibition, which has demonstrated enhanced cytoreductive action in lacrimal gland ACC cell lines [18]. Anecdotal evidence from case reports suggests that immune checkpoint inhibitors may have some benefit in lacrimal gland tumors [19,20]. However, concrete data from large-scale analyses are not available [21].

We were able to identify the occurrence of several rare types of LG tumors, on which data in the literature is very limited (Table 1). Carcinoma ex pleomorphic adenoma is one such tumor that is aggressive and carries a poor prognosis. Around 27 cases have been described so far [22]. Our analysis had 12 cases of carcinoma ex pleomorphic adenoma with an incidence of 1.79%. Oncocytomas (oxyphilic adenoma), although predominantly benign, can undergo malignant transformation into malignant oncocytoma in 5-10% of the cases, which may have an aggressive course [23]. We were able to identify seven cases (1.05%) from the NCDB database.

Drawbacks of our study include the use of observational data in which there is always a chance for confounding despite the use of propensity score weighing and multivariate model. There is a possibility that SCC may be overrepresented by tumors originating and extending from adjacent structures like the skin and sinuses.

## Conclusions

Based on the analysis from a large national database, we found that SCC and ACC are commonly encountered lacrimal gland tumors. ACC can still be considered the commonest histological subtype as the frequency of SCC and ACC was identical to each other. There is also a possibility that the SCC number may be high due to spread from adjacent sites. No difference in outcome was found between destructive and orbit sparing procedures nor the use of any form of CT or RT. Given the rarity of the disease, even large databases may not accurately represent an adequate sample. Larger population-based trials, though difficult to perform, may be needed to help providers better manage this uncommon tumor.
